# A Novel Low-Ringing Monocycle Picosecond Pulse Generator Based on Step Recovery Diode

**DOI:** 10.1371/journal.pone.0136287

**Published:** 2015-08-26

**Authors:** Jianming Zhou, Xiao Yang, Qiuyuan Lu, Fan Liu

**Affiliations:** School of Information and Electronics, Beijing Institute of Technology, Beijing, 100081, China; National Research Council, ITALY

## Abstract

This paper presents a high-performance low-ringing ultra-wideband monocycle picosecond pulse generator, formed using a step recovery diode (SRD), simulated in ADS software and generated through experimentation. The pulse generator comprises three parts, a step recovery diode, a field-effect transistor and a Schottky diode, used to eliminate the positive and negative ringing of pulse. Simulated results validate the design. Measured results indicate an output waveform of 1.88 peak-to-peak amplitude and 307ps pulse duration with a minimal ringing of -22.5 dB, providing good symmetry and low level of ringing. A high degree of coordination between the simulated and measured results is achieved.

## Introduction

Ultra-wideband narrow pulse is an important aspect of any study relating to ultra-wideband (UWB) radar and the UWB wireless communication system [1–3] owing to its high-resolution applications and simple system architecture. Numerous impulse radars have been developed for the purposes of detection, identification and scanning by analysis of transmitted and received signals [4–5]. The UWB narrow pulse can be further segregated into the classifications of step pulse, Gaussian pulse and the single-cycle pulse. All these pulses have wide frequency spectrums [6]. Step pulse and Gaussian pulse are suitable to be adopted to the receivers, because their high DC components are difficult to be emitted by the antenna. On the other hand, the single-cycle pulse can be used in the senders, owing to its lack of DC components and its minimal amount of low-frequency components.

In recent years, several studies have reported methods of generating UWB pulse, including the tunnel diode based generator [7], oscillator-based generator [8], transistor-based generator [9] and step recovery diode (SRD) based generators [1–2, 10–13]. Components should be carefully selected according to each system’s unique requirements and depending on its output pulse power and width. The SRD-based is the most popular design method for all kinds of pulse generating solution, due to its effective ability to sharpen pulse transition edge [12] and its advantage of being easy to fabricate. However many researchers have encountered the same problem, namely, a high ringing level of the pulse, which has an undesirable effect on the resolution power of sensing radars. In former research [14], we introduced a subnanosecond-wide Gaussian pulse using the shunt-connected SRD model, resulting in a ringing level of about -15dB. Through experimentation, [15] better results were attained, with a ringing level of -22dB, by using resistive stubs for suppressing the ringing tail. Nevertheless, the only shortcoming is that, the peak-to-peak pulse amplitude is very small (550mV) compared with other methods.

In this paper, our intention is to design a low-ringing monocycle picosecond pulse and to advance our former work [14, 16], with the aim of generating a signal which possesses both lower ringing and an acceptable amplitude. The remainder of this paper is structured as follows. Section II introduces the SRD model in brief. Section III describes the design circuit of the pulse generator. The test results achieved in the simulation and experiment are provided in Section IV, with conclusions provided in Section V.

## SRD Model

### 2.1 SRD structure and characteristics

**Fig 1:**
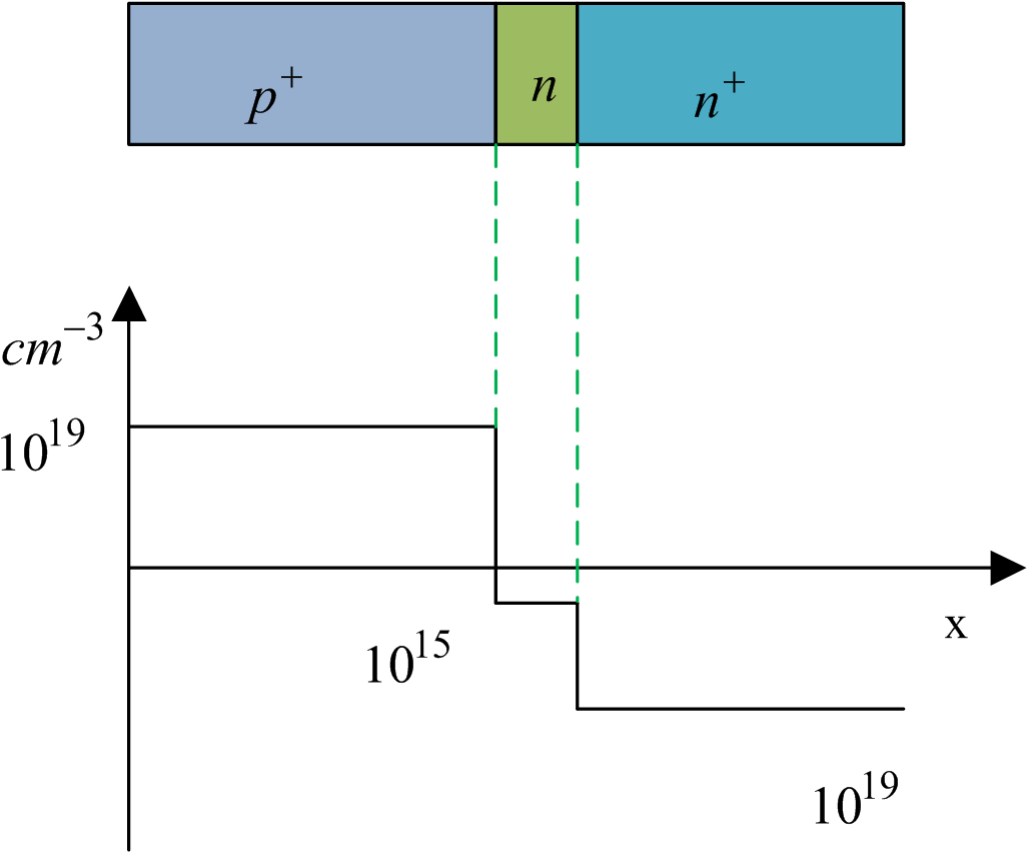
Impurity profile of SRD.

**Fig 2:**
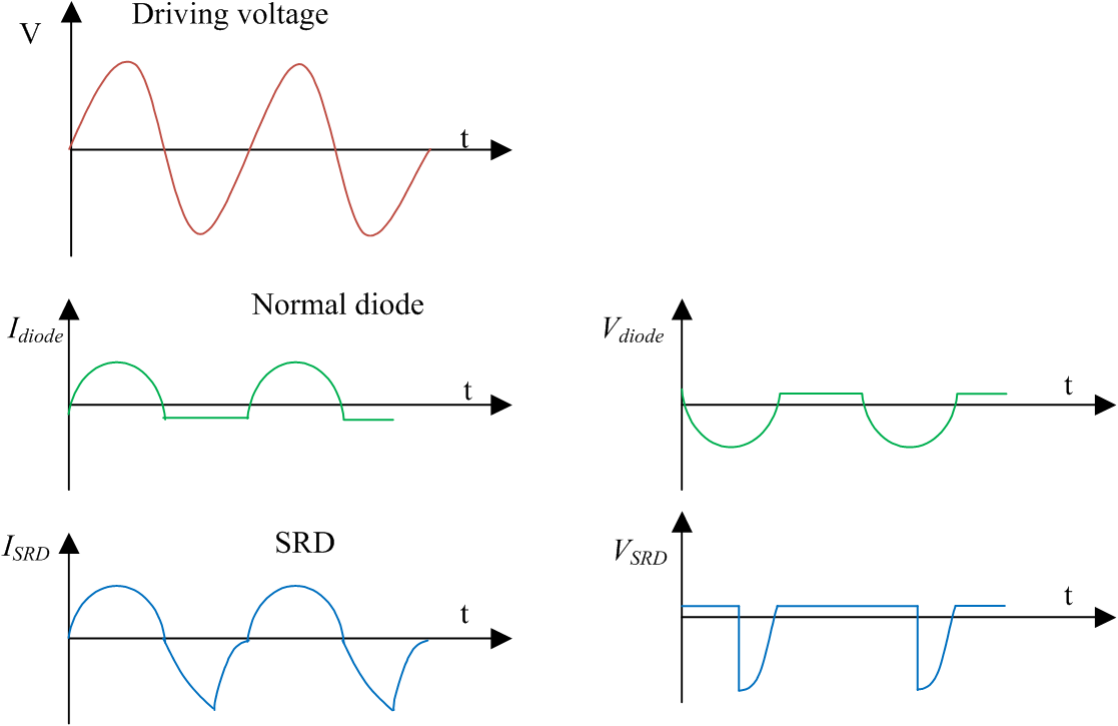
Voltage and current characteristics curves of common diode and SRD.

SRD is a PN junction diode, whose impurity exhibits an unusual distribution, as shown in Fig 1. Between the high doping P^+^ layer and the high doping N^+^ layer is a low doping N-type layer. This has a typical slowly varying junction structure. The characteristics of forward conduction and reverse cut-off, produced by the excitement of a sine wave, will be presented to an ordinary diode. In the case of the SRD, the waveforms of current and voltage are different and are shown in Fig 2. When SRD converts from forward exciting voltage to negative exciting voltage, a strong backward current flows continuously until terminating at a time instant, thus forming a steep step voltage. The narrow pulse can be generated in this way.

The principles for this phenomenon are as follows:

1. When SRD is in a positive bias, both sides of the PN junction become infused with many minority carriers. The special impurity distribution in SRD facilitates the increase in these injected minority carriers, and creating retarding fields on both sides of the junction eliciting impedance in the minority carriers’ proliferation, thus forming a concentration of minority carriers in the narrow regions near the junction. Furthermore, the increased longevity of the minority carriers is such that they are prevented from rejoining during a period of positive bias.
2. When the positive bias converts to a negative bias, minority carriers stored will flow in the opposite direction to the injection, thus forming a strong backward current. When extraction of all the minority carriers has taken place, the backward current is suddenly reduced to an extremely low level, cutting off the diode and forming the step voltage.

### 2.2 SRD equivalent circuit analysis

**Fig 3:**
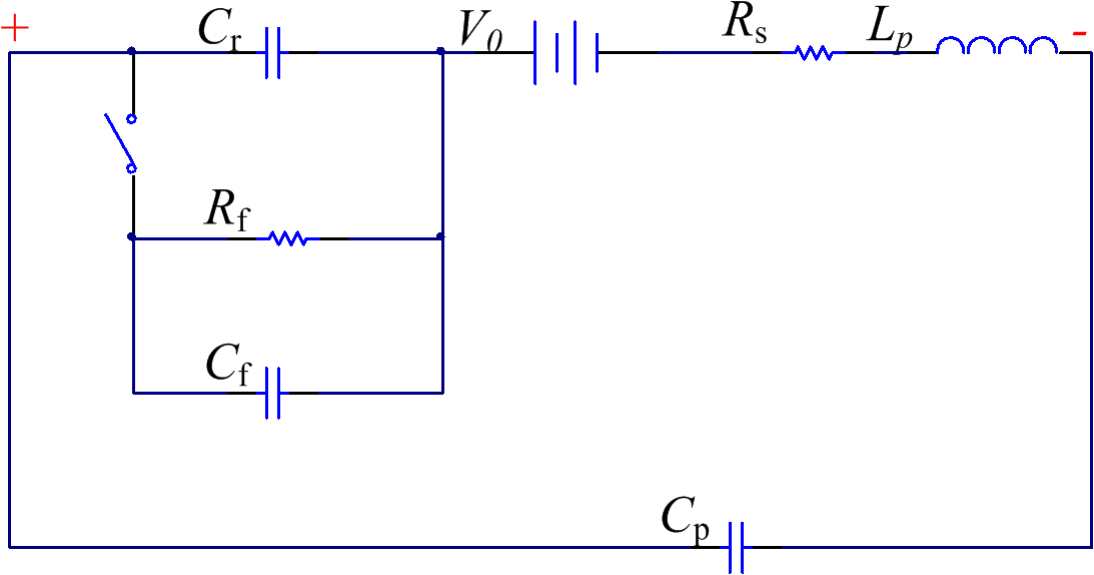
Equivalent circuit of the SRD.

The SRD equivalent circuit is depicted in Fig 3, where *C*_f_ represents the forward-bias diffusion capacitance, *C*_r_ denotes the backward-bias depletion layer capacitance, *R*_f_ represents the junction resistance of the diode, *R*_s_ denotes the series resistance of the diode, and *V*_0_, the barrier potential of the junction. The equivalent circuit shows two types of working status in the forward and backward biases. Under the forward-bias voltage, the equivalent circuit consists of the large *C*_f_ and *R*_f_, whereas the equivalent circuit consists of the small *C*_r_ under the backward-bias voltage.

Generally, the SRD parameters encompass *γ*, *C*_0_, *V*_0_ and *τ*. According to Kotzebue’s derivation:

$$\tau =R_{f}C_{f}$$ (1)

**Fig 4:**
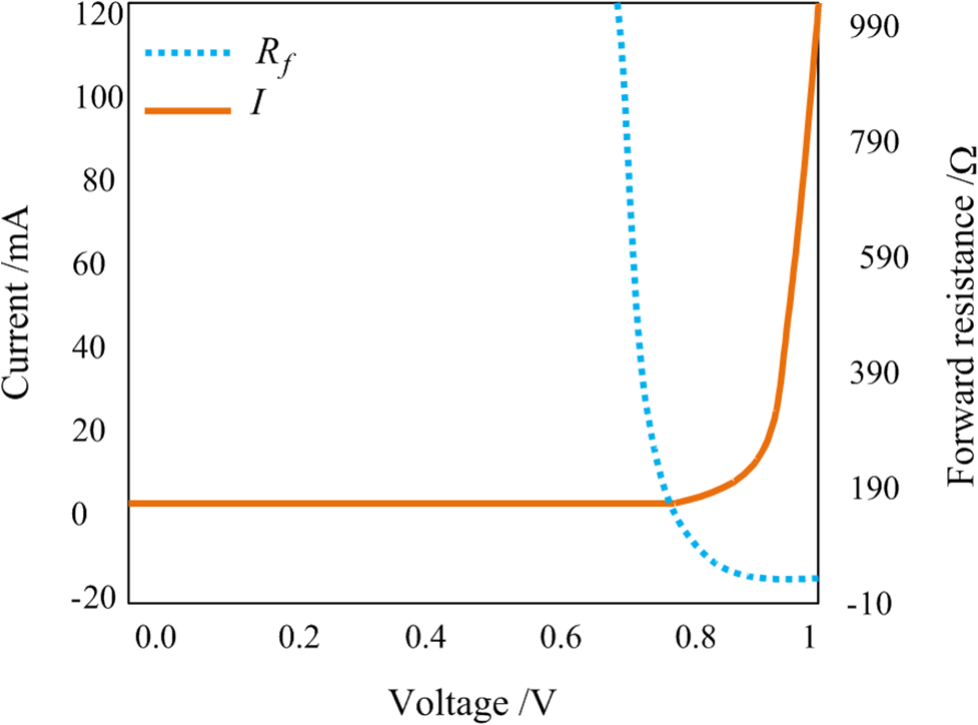
*I-V* characteristic and forward resistance changing curves.

Where, *τ* is the lifetime of the minority carrier, and *C*_f_ can be computed with *τ* and the forward on-resistance *R*_f_. The forward resistance *R*_f_ can be computed by measuring SRD’s *I*-*V* characteristics. Typical *I*-*V* characteristics and forward resistance curves are shown in Fig 4. From the figure, it can be seen that the SRD’s forward resistance *R*_f_ is computed by measuring SRD’s *I*-*V* characteristics, so the forward capacitance *C*_f_ can be computed as well. With *γ*, *C*_0_, *V*_0_ and other diode parameters, the diode’s model can be represented both easily and effectively. As the model is more accurate, consequent improvements to the hardware design are possible, along with speedier production rates in the design.

M-Pulse Microwave MP4023 is used as SRD in this paper. The parameters of τ is 15ns, *t*_t_ (the transition time) is 50ps, *C*_r_ is within 0.2~0.5pF, and *R*_s_ is 0.8Ω. The value of *C*_f_ can be calculated by the existing parameters and the *I-V* curve, as show in Fig 4. When the values of all components in the SRD equivalent circuit are known, the Spice Model can be constructed according to the relationship between SRD quantity of electricity and voltage, as shown in Eq (2) [13].

$$Q\left(V\right)=\left\{ \begin{array}{ll} C_{r}V & V\leq 0\\ \frac{C_{f}-C_{r}}{2V_{0}}{\left(V+\frac{C_{r}V_{0}}{C_{f}-C_{r}}\right)}^{2}-\frac{C_{r}{}^{2}}{2\left(C_{f}-C_{r}\right)}V_{0} & 0<V<V_{0}\\ C_{f}V-\frac{C_{f}-C_{r}}{2}V_{0} & \;V\geq V_{0} \end{array}\right.$$ (2)

**Fig 5:**
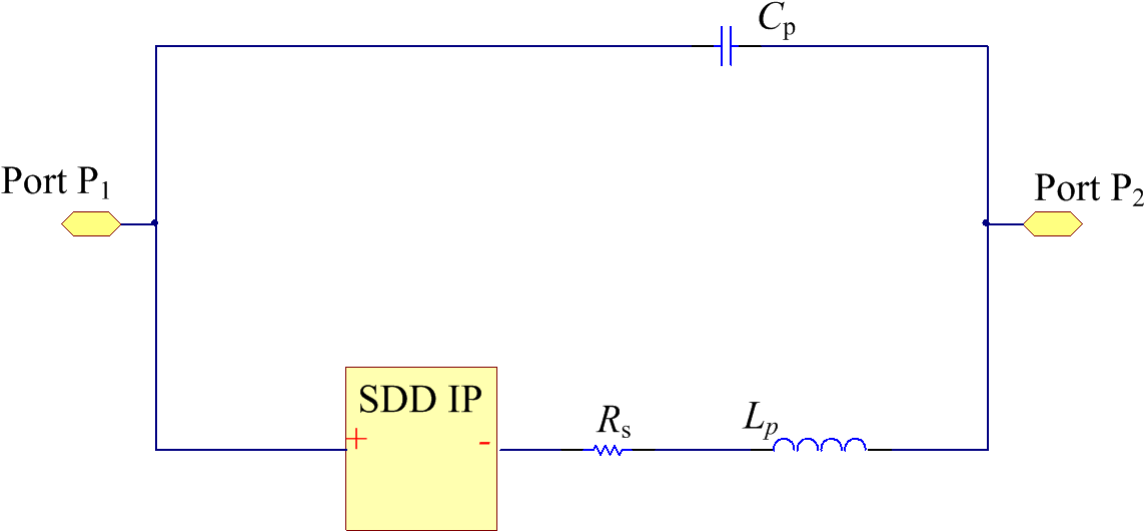
Spice model of the SRD.

Fig 5 presents the simulation diagram in ADS, where SDD denotes the relationship between current, voltage and their differentials of different ports in the N-port component. The component’s properties can be expressed by defining the relationship between ports with their algebraic expressions. By representing the voltage-current relation in Eq (1) with SDD and defining the SRD parameters of *C*_f_ and *C*_r_ that have been acquired above as the input, we can obtain the simulation model.

## Pulse Generator Circuit Design

### 3.1 Overall design

**Fig 6:**
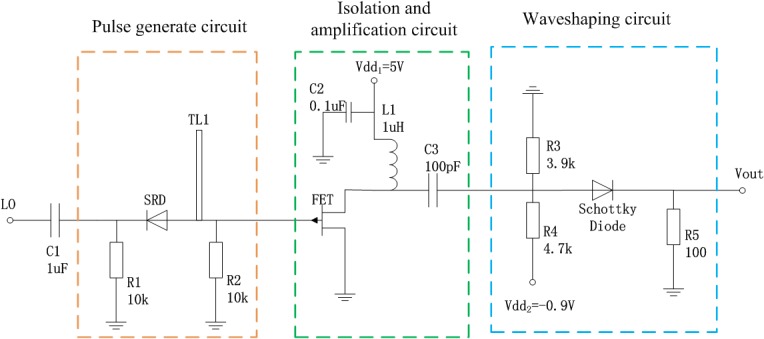
Circuit of the picosecond pulse generator.

To generate the required Gaussian narrow pulse, the circuit of the pulse generator is designed based on the prototype in [2], in which the small amplitude of output signal highlights an inevitable shortcoming. In order to overcome this issue, we add a FET module to amplify the output pulse signal. In addition, another function of the FET module is to act as a buffer between the signal generating module and the signal shaping module. Fig 6 shows the overall circuit design of the pulse generator, which consists of a pulse generating circuit, isolating and amplifying circuit, as well as the shaping circuit. The pulse generating circuit comprises a coupled circuit, match circuit, step recovery diode (SRD), and a micro-strip short-circuit line. The isolating and amplifying circuit consists of FET module, with the shaping circuit being made up of Schottky diode. A summary of parameters of main component in Fig 6 appears in Table 1, and other values for resistances and capacitances are marked in the figure.

**Table 1:** Parameters of main components of circuit.

Function	Type	Key parameter
Signal input	Square wave	10MHz
Substrate	FR-4	*ε*_*r*_ = 9.6
SRD	MP4023	*τ* = 15*ns*, *t*_t_ = 50ps, *C*_r_ = 0.2~0.5 pF
Transmission line	TL1	400 mil
Schottky diode	HSMS2820	Turn‑On Voltage 0.34 V at 1 mA
FET	FSX017LG	I_DSS_ = 55mA, C_GS_ = 0.55pF

### 3.2 Pulse generating circuit

The input periodic square wave signal excites SRD via the coupled circuit and the match circuit. The SRD self-bias circuit, consisting of the coupled circuit and *R*_L_, eliminates the need for the extra DC biasing circuit, thereby reducing the overall size of the circuit, exercises good control over charge storage and ensures an optimum output of the pulse at high amplitude. The match circuit is used to achieve maximum transmission of power, owing to the input impedance of SRD being within the range 10–20Ω, while the input impedance of the entire system is 50Ω. Furthermore, an extremely fast step pulse can be generated after the signal passes through SRD. In addition, it will yield a narrow pulse with a long tail and width by subtracting the forward-propagation step pulse from the reflected step pulse after passing through the micro-strip short-circuit line. Therefore the process needs to be carried out by the subsequent circuits.

The following matter requires special consideration during the design process. The length of the micro-strip short-circuit line is determined by the pulse width, as shown in Eq (3).

$$t_{A}=\frac{2l}{v}$$ (3)

Where, *t*_*A*_ denotes the pulse width, *l* represents the length of the micro-strip short-circuit line, and $v=\frac{c}{\sqrt{\varepsilon_{\text{re}}}}$(c is the speed of light), *ε*_re_ is effective dielectric constant. According to Eq (3), the length of the short-circuit line can be acquired after the pulse width *t*_*A*_ is achieved.

### 3.3 Isolating and amplifying circuit

The function of FET module has two aspects, the first and more important role is to amplify the pulse signal, and the other is to isolate the pulse generating circuit from the pulse shaping circuit, which can be seen in the Fig 6. The working principle of the amplifier circuit is as follows: the positive DC bias voltage is applied to the drain electrode of FET by V_dd1_, and the pulse signal generated by MP4023 is connect to the grid electrode. Before the narrow pulse coming, FET is functioning in a saturation zone and fails to work. However, when the negative pulse arriving, FET works in the linear amplification zone due to the negative bias voltage, and amplifies the narrow pulse signal. After being amplified by FET, the narrow pulse will be of larger amplitude and convert from a negative pulse to a positive pulse.

### 3.4 Function of Schottky Diode

The ringing level of pulse signal is further reduced by the Schottky diode in the shaping circuit, serving as a high-speed switch, which only allows a pulse greater than a certain threshold to pass. This threshold is determined by the DC bias voltage *V*_d_. The negative bias voltage can lower the pulse form overall and theoretically eliminate any positive and negative ringing. Despite this, it is impossible as the switch time of the Schottky diode could not be equal to zero. So the phenomenon of ringing still exists in the output and the value of *V*_d_ is determined by the value of the ringing. In this design, the *V*_d_ is set by -0.9V, as shown in Fig 6.

## Simulation and Experimental Results

**Fig 7:**
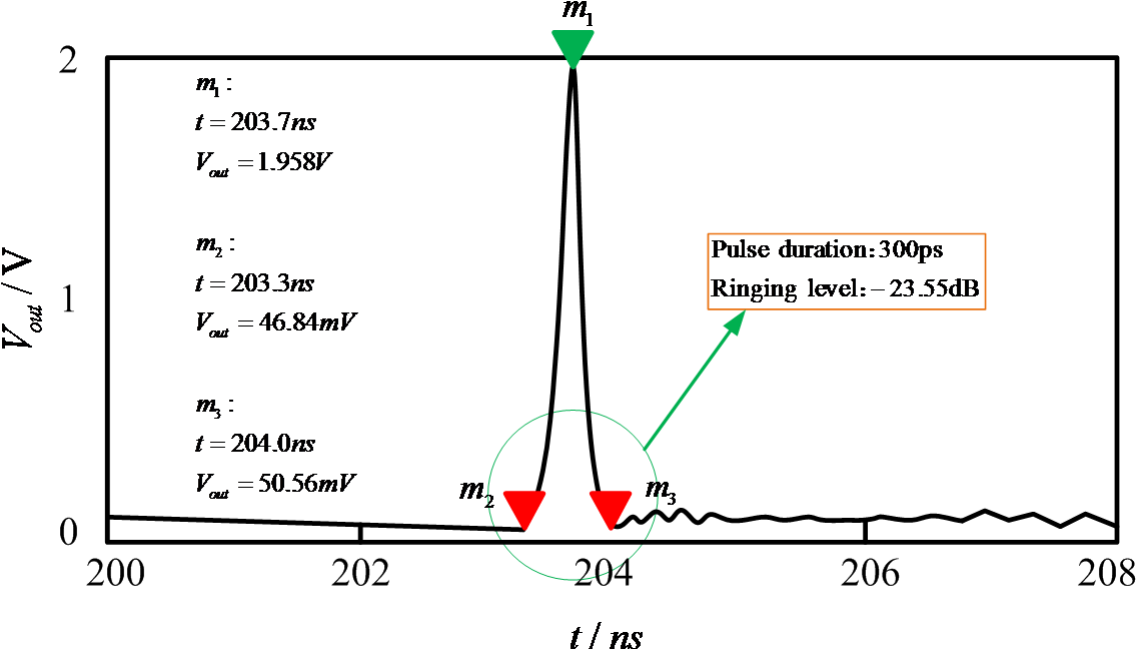
Simulation of the output pulse.

According to the SRD model and designed circuit as shown in Fig 6, we simulate the pulse generator in ADS software. Fig 7 shows the waveform of the output pulse in simulation. In the figure, the SRD model has been applied to the pulse generator. One can see that the pulse width is about 300ps and the pulse amplitude is about 1.96 V. The waveform in the simulation is satisfactory because nearly all tails and ringing have been eliminated.

The detailed ringing level *R*_*l*_, can be calculated by Eq (4), which was reduced to -23.55dB, with the peak-to-peak amplitude of ringing at about 130mV.

$$R_{l}=20\;\text{log}\frac{Amplitude\;of\;ringing_{peak-to-peak}}{Amplitude\;of\;pulse_{peak-to-peak}}$$ (4)

**Fig 8:**
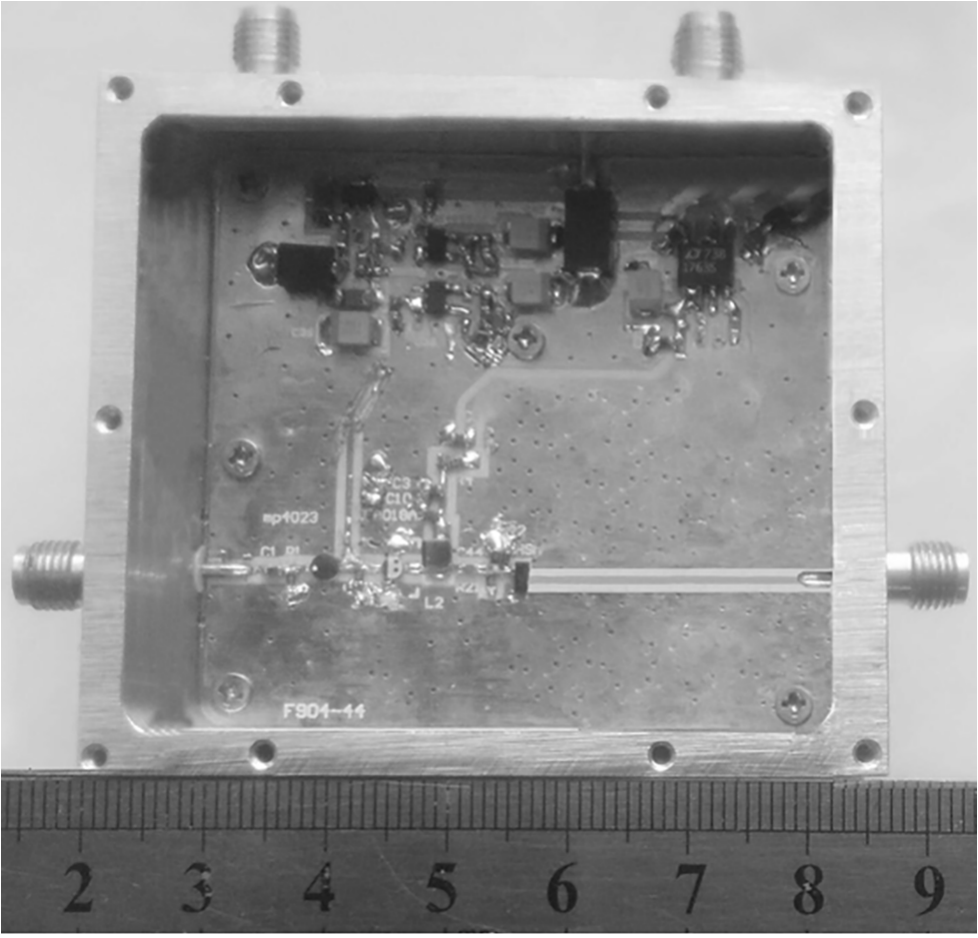
Prototype of designed pulse generator.

**Fig 9:**
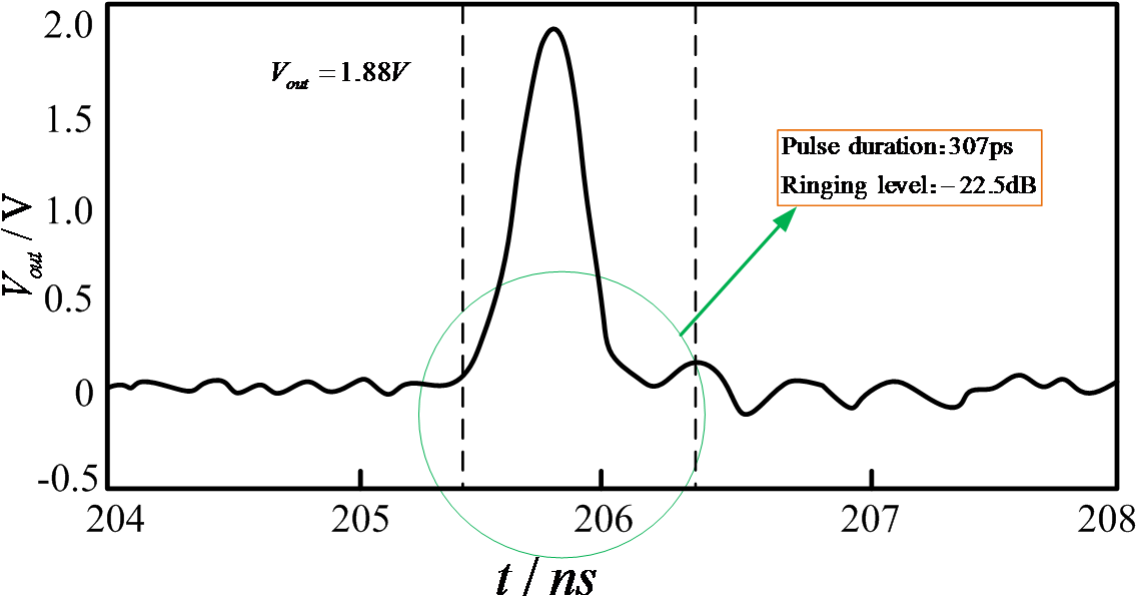
Measured output pulse of the generator.

The circuit of the pulse generator is made utilizing Teflon boards. Its permittivity is 9.6, its density 0.8mm, and the micro-strip line is 50Ω. The design of the circuit structure is compact, as shown in Fig 8. The input signal is 10 MHz rectangular wave, and the waveform is monitored by Lecroy Wave Master 8600A 6GHz DSO oscilloscope as shown in Fig 9. The pulse amplitude is 1.88 V and the pulse width is 307ps. A comparison between the experimental result and the simulation result demonstrates that the pulse amplitude in the test is smaller than that in the simulation, and the pulse ringing in the test is greater than that in the simulation. The ringing level of pulse is -22.5dB, with the peak-to-peak amplitude of ringing being 135mV. Overall the two basic results are consistent. Moreover, the generated narrow pulse is highly symmetric and without huge tails and ringing.

Furthermore, in order to evaluate the performance of the proposed pulse generator, a comparison of various UWB pulse generators constructed from discrete components which have been used in recent research has been given as shown in Table 2. It can be inferred that our design has a very low ringing level [2, 10, 12, 13] and narrow pulse [10, 12, 13]. In [2], the circuit was more complicated and exhibited an increase in power consumption owing to the second DC bias Schottky Diode. It can be said however, compared to the literature noted in [12, 14], which also possess relatively low ringing results, that our designed pulse achieves an acceptable amplitude.

**Table 2:** Comparisons with pulse generations in literatures.

Reference	Pulse amplitude /V	Pulse duration /ps	*R*_l_ /dB
[1]	0.7	350	-20.9
[2]	0.4	300	-17
[10]	0.2	600~780	-20
[12]	0.87	170	-22
[13]	1.8~1.85	1000	-17.15
[14]	15	400	-15.56
[15]	0.55	320	-22
This work-S	1.96	300	-23.55
This work-E	1.88	307	-22.5

## Conclusion

A SRD model is constructed by using ADS simulation software in this paper. The researchers also design, simulate, construct and test a UWB picosecond pulse generator. The proposed generator proves to be structurally compact, cheap to build and easy to implement. The width and amplitude of the tested pulse are 307ps and 1.88V respectively. The test results are consistent with the simulation results. The proposed generator can therefore be adapted to practical applications and be introduced to either UWB pulse emitting components or down-conversion sampling receivers. We conclude that this model has great potential to be widely used.
